# Implementation strategies and outcomes of school-based programs for adolescent suicide prevention: A scoping review protocol

**DOI:** 10.1371/journal.pone.0284431

**Published:** 2023-05-04

**Authors:** Belén Vargas, Pablo Martínez, Scarlett Mac-Ginty, Tamara Hoffmann, Vania Martínez

**Affiliations:** 1 Millennium Nucleus to Improve the Mental Health of Adolescents and Youths Imhay, Santiago, Chile; 2 Doctoral Program in Psychotherapy, Faculty of Medicine and Faculty of Social Sciences, Universidad de Chile and Pontificia Universidad Católica de Chile, Santiago, Chile; 3 Faculté de Médecine et des Sciences de la Santé, Université de Sherbrooke, Québec, Canada; 4 Centre de Recherche Charles-Le Moyne, Québec, Canada; 5 Millennium Institute for Research in Depression and Personality MIDAP, Santiago, Chile; 6 Faculty of Dentistry, Universidad de Chile, Santiago, Chile; 7 King’s College London, Department of Health Service and Population Research, Institute of Psychiatry, Psychology & Neuroscience, London, United Kingdom; 8 CEMERA, Faculty of Medicine, Universidad de Chile, Santiago, Chile; Xiamen University - Malaysia Campus: Xiamen University - Malaysia, MALAYSIA

## Abstract

**Objective:**

This scoping review aims to identify and map the empirical literature on the implementation strategies and outcomes of school-based programs for adolescent suicide prevention (SBASP).

**Introduction:**

School-based programs are preferred interventions for preventing suicide in adolescents, and their effectiveness has been well-systematized in several reviews. Implementation research is a growing field for prevention programs, making it possible to understand the nature of success or failure outcomes and maximize intervention benefits. However, there is a knowledge gap in the implementation research applied to adolescent suicide prevention in the educational context. We conduct a scoping review to provide the first overview of the scope of implementation research applied to adolescent suicide prevention programs in the school setting to know what implementation strategies and outcomes are reported by these programs and how they are evaluated.

**Methods:**

The proposed scoping review will be conducted following six stages, including the definition of objectives. Studies must be empirical and address implementation strategies or implementation outcomes of school-based programs for adolescent suicide prevention. Studies that focused exclusively on clinical efficacy or effectiveness evaluation will be excluded. A preliminary search of PubMed was conducted to refine the initial search strings, followed by a final search of several other electronic databases. Finally, a gray literature search will identify unpublished literature and reduce location bias. There will be no limits to a specific date. Two independent reviewers will screen, select, and extract the retrieved records. The results will be presented using tabular forms and a narrative summary with attention to the review objectives and research questions and their implications for research and practice of school-based programs for adolescent suicide prevention.

## Introduction

Adolescent suicidal behavior remains a global public health concern [1], especially in the uncertain territory of the COVID-19 pandemic and its consequences on mental health [2]. According to global statistics, suicide is the fourth leading cause of death in people aged 15–19, although the rates have decreased since 2008 [3]. Suicide causes 4.06% of healthy life-year losses for this age group [4].

School contexts offer unique opportunities to implement adolescent suicide prevention programs [5], with venues where most interventions for this population are conducted [6]. Generally, school-based programs for adolescent suicide prevention (SBASP) involve one or more components, such as gatekeeper training, suicide awareness education, help-seeking promotion, screening, and social-emotional learning interventions [7]. Evidence for their effectiveness in suicide prevention is inconclusive. Some relevant outcomes are increased suicide awareness and reduced suicide behavior stigma; few studies have demonstrated direct effects on suicide behavior reduction and psychological wellness improvement [8].

However, effectiveness studies appear to be insufficient to assess the outcomes of suicide prevention programs. Recommendations for effective SBASP emphasize considering contextual factors, implementation strategies, and delivery characteristics as critical elements in their design [9]. Evidence suggests that the implementation conditions and settings operate as effectiveness moderators [10]. The World Health Organization (WHO) indicated that suicide prevention program evaluations should also consider implementation indicators in their most recent suicide prevention guidelines [11]. More studies are necessary to understand the heterogeneity of SBASP effectiveness across settings, and what types of interventions work for specific institutional cultures and participants [12]. Research centered on the implementation process of SBAPS can contribute to identifying the optimal strategies for implementing these interventions.

Implementation science studies methods, processes, and core elements associated with successfully integrating evidence-based practices (EBPs) into real-world settings to improve health services and care [13]. Incorporating implementation effectiveness into program evaluations makes assessing interventions’ internal and external validity possible, helping determine whether their failure or success was due to a lack of effectiveness in the new context, how they were delivered, or both. An exemplary implementation makes it possible to maximize the benefits for participants, for example, in prevention programs [14].

Implementation occurs through various strategies defined as methods, techniques, or activities to put into practice and sustain evidence-based innovations; the strategies are the implementation *how*. To understand the implementation process, the effectiveness of its strategies, and implementation success, it is necessary to measure its outcomes [14–16].

This field has generated solid frameworks to guide research, including the Implementation outcomes framework (IOF) developed by Proctor and colleagues [15]. This heuristic taxonomy organizes implementation outcomes around eight core constructs: acceptability, adoption, appropriateness, feasibility, fidelity, implementation cost, penetration, and sustainability. The IOF operationalizes implementation outcomes, emphasizing differences and interrelations between intervention and client outcomes [17]. For its part, the Expert Recommendations for Implementing Change (ERIC) Project developed a taxonomy of implementation strategies, proposing a common language of 73 categories based on the consensus of researchers and implementers of interventions in healthcare [18]. Recently, this taxonomy was adapted in the School Implementation Strategies, Translating ERIC Resources (SISTER) project through a methodology similar to that used in the initial construction, resulting in 75 discrete categories of implementation strategies for school contexts [19].

### Problem statement

There remains a gap between research and practice in school-based prevention. Implementation research needs to advance from a barriers and facilitators approach to systematic studies focusing on practices and their quality, striving to determine how they can increase positive student outcomes and accelerate and enhance the adoption of EBPs in schools [19–21]. Focus on the implementation process improves the potential impact of SBAPS and accelerates the translation of research into practice; implementation science applied to suicide prevention could be an opportunity to implement effective life-saving interventions rapidly [22].

However, there is a lack of research on the implementation effectiveness of suicide prevention interventions [23].

We conducted a preliminary search of PROSPERO, PubMed, the Cochrane Database of Systematic Reviews, and JBI Evidence Synthesis, finding the absence of current or in-progress scoping reviews or systematic reviews on the topic. Nevertheless, several studies reported implementation outcomes of SBASP, such as universal screening for suicide risk [24], awareness programs [25], and gatekeeper training [26], and used various informants and methodological approaches [23, 27, 28]. Given the limited knowledge systematized on the implementation of SBASP, the present protocol proposes the first review in the field with the aim to identify and map the empirical literature on the implementation strategies and implementation outcomes of SBASP. Through a scoping review, we expected to identify how implementation research is conducted on SBASP and the type of existing evidence in this field. The results could inform the study and design of new interventions to reduce suicide behavior in young people and strengthen ongoing programs.

### Review question

In line with the aim of this review, our research questions are:

What implementation strategies and outcomes are reported by school-based programs for adolescent suicide prevention (SBASP)?

How are the implementation strategies and outcomes of SBASP evaluated?

What implementation outcomes are obtained in accordance with the SBASP’s levels of prevention and program components?

## Methods

**Fig 1:**
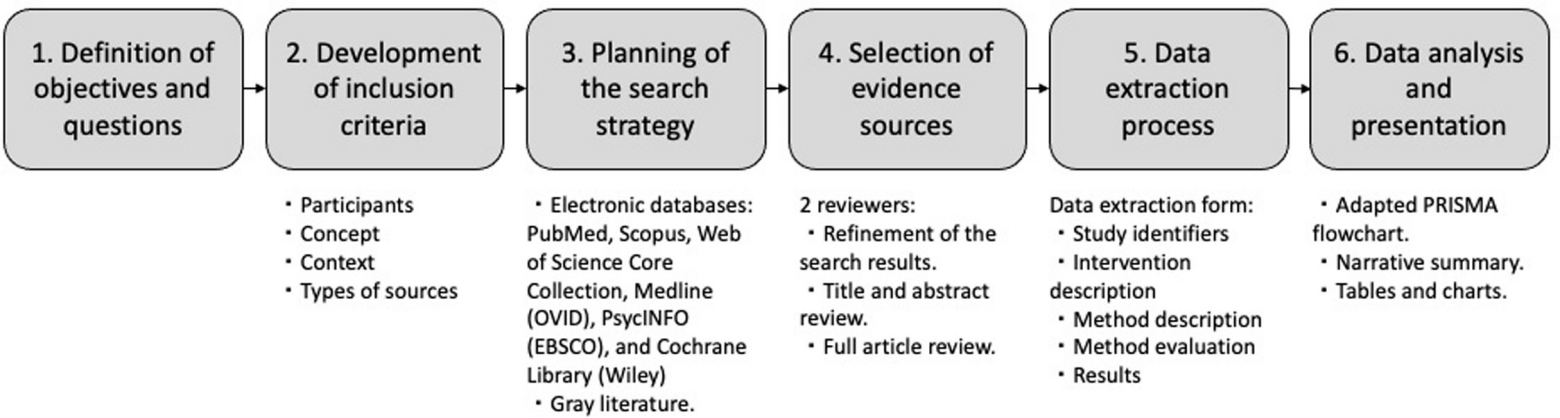
Scoping review stages.

A scoping review is the best way to explore the extent of the literature, map and summarize the findings, and inform future research in an emerging field. Scoping reviews incorporate a wide range of evidence and research methodologies through an explicit, rigorous, and replicable method [29, 30]. The proposed scoping review will be conducted following standard methods and the JBI Manual for Evidence Synthesis [29], comprising six stages: 1) definition of objectives and questions, 2) development of inclusion criteria, 3) planning of the search strategy, 4) selection of evidence sources, 5) data extraction process, and 6) data analysis and presentation (Fig 1).

The review protocol was registered in the Open Science Framework (https://osf.io/v8gbn/) [31] and is reported in agreement with the Preferred Reporting Items for Systematic Reviews and Meta-analyses Extension for Scoping Reviews (PRISMA-ScR) (S1 Checklist) [30]. Ethical approval is not required for this study.

### Inclusion criteria

#### Participants

Based on the WHO adolescent criteria, this review will consider studies that include school students between 10 and 19 years [11]. Studies with participants outside this age range will be considered if the mean age of the sample is within the inclusion criterion.

#### Concept

To be included in the review, studies should evaluate the implementation of SBASP. The clinical effectiveness or efficacy of these programs must have been proven; alternatively, programs must have been EBPs. To be eligible, studies must report implementation strategies used and/or implementation outcomes, the latter according to the IOF [15]. The studies could use various instruments or data sources from several approaches to evaluate the implementation. Studies focusing only on the efficacy, effectiveness, and clinical outcomes of intervention will be excluded since these studies typically do not incorporate implementation outcomes such as the ones we intend to document. Likewise, we will exclude programs whose main objectives do not include suicide behavior prevention, instead featuring it as a secondary target or an indirect result since this is out of the scope of our review. Table 1 provides a brief definition of the key concepts of the protocol.

**Table 1:** Definitions of key concepts scoping review.

Concept	Definition
School-based programs for adolescent suicide prevention [SBASP]	An intervention delivered in an educational setting aimed at reducing the occurrence of suicidal behavior in adolescents. SBASP can be universal, selective, or indicated and include one or more components like gatekeeper training, curriculum-based education, and identification of students’ suicide risk [8, 12]. The intervention’s effectiveness/efficacy must have been proven (evidence-based program); alternatively, they must have been developed by integrating or adapting EBPs (evidence-informed program).
Implementation strategies	Methods, techniques, or activities to put into practice and sustain an intervention or program, focusing on factors that influence their implementation [16]. A broad range of possible strategies can be organized into discrete categories for tracking and reporting. These categories include providing interactive assistance, adapting and tailoring interventions to a given context, developing stakeholder relationships, delivering training or education, and offering technical support and financial strategies, among others [18, 19].
Implementation outcomes	Defined as "the effects or deliberate and purposive actions to implement new treatments, practices, and services" [15]. They are indicators of implementation success. According to the Implementation outcomes framework, these outcomes are acceptability, adoption, appropriateness, feasibility, fidelity, implementation cost, penetration, and sustainability [15].

#### Context

The interventions must be implemented in the school context. There will be no restrictions on geographical location, provider staff type, delivery modes, length, or sociocultural characteristics (e.g., gender, ethnicity, racialized groups).

#### Types of sources

Published and unpublished primary sources of evidence and reviews will be included. In line with the nature of implementation science, a broad range of research designs will be considered for inclusion. Quantitative, qualitative, or mixed-method study designs will be considered, including experimental and quasi-experimental study designs, case reports, and hybrid effectiveness-implementation studies. Additionally, we will consider literature reviews, such as systematic reviews, scoping reviews, and meta-analyses, to identify primary sources. Gray literature will be included to maximize the number of potential studies eligible for inclusion, reduce publication bias, and gain knowledge from interventions implemented by governments, international agencies, or non-governmental organization initiatives (e.g., reports, policy briefs). Textbooks, program evaluations, toolkits, and guidelines will also be considered. This review will exclude documents comprising non-empirical reports, such as protocols, methodological papers, dissertations, newsletters, or opinion papers, since these records do not present original results or may not have been peer-reviewed. Theses and conference proceedings will also be excluded.

### Search strategy

In line with the JBI recommendations, a four-step search strategy will be utilized. First, a preliminary and limited search will be conducted on PubMed, using initial search strings combined with a series of free-text terms related to the concepts examined in this review: a) suicide, b) adolescents, c) prevention, d) school-based program, e) implementation, f) implementation strategies, and g) implementation outcomes (S1 Appendix). The retrieved papers’ titles, abstracts, and keywords will be analyzed to refine the search strings. The following step will be a second search using the final search strings adapted for each of the following electronic databases: PubMed, Scopus, Web of Science Core Collection, Medline (OVID), PsycINFO (EBSCO), and the Cochrane Library (Wiley). Third, gray literature searches will be performed through the websites Open Grey, Grey Matters, and Google Scholar, using the Chrome browser in anonymous mode to reduce geographical bias. Finally, we will review the systematic reviews’ reference list to find additional studies.

If only the abstract of a given item is available, the author will be contacted to obtain full access to the paper. This review will include articles published in English or Spanish, and there will be no limits to the specific publication time.

### Study selection

The study selection will be completed in three stages: 1) refinement of the search results, 2) title and abstract review, and 3) full article review. All retrieved records will be imported into Endnote v.X9.3.3 (Clarivate Analytics, Pennsylvania, EE. UU.) to detect duplicates and citations without abstracts, which will be manually removed. Before screening and selecting studies, we will test the eligibility criteria and their definition in ten percent of titles and abstracts. If necessary, the team will discuss discrepancies and modify the eligibility criteria definitions to reduce ambiguity and error. Once the team achieves at least 75% agreement, the selection process will resume.

Two independent reviewers will screen all the titles and abstracts using Rayyan (Qatar Computing Research Institute, Doha, Qatar) to check for eligibility. If an article’s relevance is unclear from the title or abstract, it will be retained for further review in the next stage. Finally, the full texts of the selected studies will be retrieved and imported to Rayyan. The same reviewers will independently assess the full text using the eligibility criteria for final inclusion.

Discrepancies found in stages 2 and 3 will be resolved through discussion between the reviewers and involve a third reviewer if needed.

In each stage, the articles excluded and the reasons for their exclusion will be reported. If we lack accurate information to judge the eligibility of an article, we will contact the study authors for further assistance.

### Data extraction

Data will be extracted from papers included in the scoping review by two independent reviewers using a data extraction form. The data extraction form will be developed for this review based on the Standards for Reporting Implementation Studies (StaRI) [32]. Five main data-coding categories will be included: 1) study identifiers, 2) intervention description, 3) method description, 4) method evaluation, and 5) results. Implementation strategies and implementation outcomes will be coded using the taxonomies developed by Cook et al. [19] and Proctor et al. [15], respectively (S2 Appendix). A codebook was developed to aid in data extraction; it contains definitions and examples for the concepts in each major data-coding category. The codebook is available in the Open Science Framework protocol registration [31].

Two reviewers will pilot the draft form for 15% of the selected papers to ensure reliability and accuracy. Disagreements will be solved through consensus, and the draft form will be modified before data extraction from all studies. One reviewer will conduct data extraction, and a second will verify the extraction. Any disagreements will be resolved through discussion and the input of a third reviewer when necessary. The authors will be contacted to clarify the results or obtain additional information if needed.

### Data analysis and presentation

We will provide a narrative and quantitative description of the search process results and an inclusion decision flowchart adapted from the PRISMA flowchart [33]. Based on the review questions, the data will be summarized in tabular form, and descriptive statistics will be reported (e.g., percentages and frequencies). The findings will be grouped under the main conceptual categories used for data extraction, explaining each. Finally, the review results will be presented through a narrative summary accompanied by tables and charts describing how the results relate to the review objectives and research questions.

## Discussion

Before the COVID-19 pandemic, suicide was among the leading causes of adolescent death, surpassed only by traffic accidents, interpersonal violence, and tuberculosis, causing 4.06% of healthy life-years losses and around 35.999 deaths per year for this age group [3, 4, 34].

The impact of COVID-19 on mental health and socioeconomic determinants has a potential intensifier of suicidal risk, especially in young people and high at-risk populations; therefore, efforts to deliver effective suicide prevention strategies should be a priority [35]. SBAPS are recommended preventive intervention that allows easy and continuous access to this population [5, 6]. However, it is necessary to identify the best ways to implement it in real-world contexts, considering the effects of implementation conditions on the intervention effectiveness, such as delivery characteristics or contextual factors [9, 10]. Implementation science applied to suicide prevention is a growing field that optimizes the installation, relevance, and scale-up of EBPs in real-world contexts [22], increasing the external validity, applicability, and usefulness of the findings [36]. Implementation studies offer contextual keys for future designs and evaluation programs and multilevel suicide prevention models; implementation evaluations are particularly valuable for lessons learned, considering that suicide prevention interventions require significant amounts of time and resources for their design, implementation, and sustainability [37]. Applying implementation science to youth mental health interventions is possible to know how and why they work or not, which could allow increasing their impact to face the post-COVID-19 era [38].

We conduct a scoping review to provide the first overview of the scope of implementation research applied to adolescent suicide prevention programs in the school setting to know what implementation strategies and outcomes are reported by these programs and how they are evaluated. In addition, we expect to distinguish the implementation strategies and outcomes employed according to the components and prevention level of evaluated programs. We chose this type of evidence synthesis due to its appropriateness to map and summarize an emerging field [29, 30] as implementation science applied to SBASP.

Once completed, our review will provide a better understanding of the emerging field by examining how the implementation evaluation is conducted, which implementation strategies are most reported in the school context, and the most used implementation results to evaluate their success. In addition, our study may be a precursor to further focused syntheses of evidence that can address more specific research questions. The results may encourage research on more in-depth evaluations of interventions to reduce adolescent suicide in the school setting, including their implementation. The findings may improve the design of new interventions and their translation to different contexts; thus, the review will be disseminated to policymakers, stakeholders, and researchers from health and education.

## Limitations

The significant limitations of our review are the need for a formal assessment of study quality and the unstructured character of results synthesis, which is characteristic of a scoping review. The substantial variation we anticipate in study designs and concepts evaluated precludes such formalisms, as we expect to find everything from qualitative studies to randomized controlled trials. However, our review will establish conceptual boundaries and knowledge gaps to inform future synthetic efforts, more direct in scope (e.g., systematic reviews, meta-analysis).

Another shortcoming is the language limiters, which restrict us to English or Spanish literature in which the reviewers are fluent. Still, we assume this criterion will not significantly affect the findings since the journals that publish content relevant to implementation science are mainly in English [39].

Any amendments made during the study will be included in the protocol registry and are reported in the results manuscript.

## Supporting information

S1 Checklist PRISMA-ScR Preferred Reporting Items for Systematic reviews and Meta-Analyses extension for Scoping Reviews checklist. (DOCX)

S1 Appendix Preliminary PubMed search strategy. (DOCX)

S2 Appendix Categories data extraction form. (DOCX)
