# Supplementary material for: Implementation strategies and outcomes of school-based programs for adolescent suicide prevention: A scoping review protocol
Source: PLoS One. 2023 May 4;18(5):e0284431. doi: 10.1371/journal.pone.0284431 (PMC10159337; doi:10.1371/journal.pone.0284431)
Supplement: S1 Appendix — (DOCX) [file pone.0284431.s002.docx]

# S1 Appendix: Preliminary PubMed search strategy

Search conducted on October 28, 2021

| **Search** | **Query** | **Records retrieved** |
| --- | --- | --- |
| #1 | ("suicid*"[Title/Abstract] OR "suicide attempt*"[Title/Abstract] OR "suicide behavior*"[Title/Abstract] OR "suicide ideation"[Title/Abstract] OR "self-harm"[Title/Abstract] OR "self injur*"[Title/Abstract] OR “suicide risk” [Title/Abstract] OR "Suicide"[MeSH Terms]) | [108,086](https://pubmed.ncbi.nlm.nih.gov/?term=%28%22suicid%2A%22%5BTitle%2FAbstract%5D+OR+%22suicide+attempt%2A%22%5BTitle%2FAbstract%5D+OR+%22suicide+behavior%2A%22%5BTitle%2FAbstract%5D+OR+%22suicide+ideation%22%5BTitle%2FAbstract%5D+OR+%22self+harm%22%5BTitle%2FAbstract%5D+OR+%22self+injur%2A%22%5BTitle%2FAbstract%5D%29+OR+%28%22Suicide%22%5BMeSH+Terms%5D%29&sort=relevance&ac=no) |
| #2 | ("prevent*"[Title/Abstract] OR "early intervention*"[Title/Abstract] OR "Primary Prevention"[Title/Abstract] OR "screen*"[Title/Abstract] OR "detection"[Title/Abstract] OR "gatekeeper*"[Title/Abstract] OR "literacy"[Title/Abstract] OR "awareness"[Title/Abstract] OR ("suicide/prevention and control"[MeSH Terms] OR ("intervention*"[Title/Abstract] OR "training train*"[Title/Abstract] OR "program model*"[Title/Abstract] OR "system*"[Title/Abstract] OR "strateg*"[Title/Abstract] OR "practice*"[Title/Abstract] OR "tool*"[Title/Abstract] OR "guideline*"[Title/Abstract] OR "plan*"[Title/Abstract] OR "education program*"[Title/Abstract] OR "preparation"[Title/Abstract] OR "educat*"[Title/Abstract]) | 10,534,108 |
| #3 | ("Adolescent"[MeSH Terms] OR "adolesce*"[Title/Abstract] OR "student*"[Title/Abstract] OR "teens"[Title/Abstract] OR "teenager*"[Title/Abstract] OR "youth*"[Title/Abstract] OR "young people"[Title/Abstract]) | 2,471,872 |
| #4 | ("School-based"[Title/Abstract] OR "school*"[Title/Abstract] OR "teacher*"[Title/Abstract] OR "school mental health"[Title/Abstract] OR "school health service*"[Title/Abstract] OR "School Health Services"[MeSH Terms]) | 341,314 |
| #5 | #1 AND #2 AND #3 AND #4 | 2,987 |
| #6 | ("implement*"[Title/Abstract] OR "Implementation Science"[Title/Abstract] OR "disseminat*"[Title/Abstract] OR "D&I"[Title/Abstract] OR "translat*"[Title/Abstract] OR "transfer*"[Title/Abstract] OR "knowledge transfer"[Title/Abstract] OR "facilitator*"[Title/Abstract] OR "barrier*"[Title/Abstract] OR "contextual factor*"[Title/Abstract] OR "formative evaluation"[Title/Abstract] OR "implementation research"[Title/Abstract] OR "process evaluation"[Title/Abstract] OR "Implementation Science"[MeSH Terms] OR ("implementation strateg*"[Title/Abstract] OR "implementation plan*"[Title/Abstract] OR "implementation process*"[Title/Abstract] OR "implementation model*"[Title/Abstract] OR "framework*"[Title/Abstract] OR "scale up"[Title/Abstract] OR "scaling up"[Title/Abstract]) OR ("acceptability"[Title/Abstract] OR "satisfaction"[Title/Abstract] OR "agreeable"[Title/Abstract] OR ("Adoption"[Title/Abstract] OR "knowledge translation"[Title/Abstract] OR "uptake"[Title/Abstract] OR "intention to adopt"[Title/Abstract]) OR ("Appropriateness"[Title/Abstract] OR "applicability"[Title/Abstract] OR "compatibility"[Title/Abstract] OR "perceived fit"[Title/Abstract] OR "fitness"[Title/Abstract] OR "sustainability"[Title/Abstract] OR "relevance"[Title/Abstract] OR "usefulness"[Title/Abstract]) OR ("Feasibility"[Title/Abstract] OR "transferability"[Title/Abstract] OR "applicability"[Title/Abstract] OR "practicability"[Title/Abstract] OR "workability"[Title/Abstract]) OR ("Fidelity"[Title/Abstract] OR "adherence"[Title/Abstract] OR "integrity"[Title/Abstract] OR "quality delivery"[Title/Abstract] OR "accuracy"[Title/Abstract]) OR ("Penetration"[Title/Abstract] OR "integration"[Title/Abstract] OR "infiltration"[Title/Abstract]) OR ("sustain*"[Title/Abstract] OR "maintenance"[Title/Abstract] OR "long-term implementation"[Title/Abstract] OR "routinization"[Title/Abstract] OR "durability"[Title/Abstract] OR "institutionalization"[Title/Abstract] OR "capacity building"[Title/Abstract]) OR ("Cost"[Title/Abstract] OR "expense"[Title/Abstract] OR "budget"[Title/Abstract] OR "marginal cost"[Title/Abstract] OR "cost-effectiveness"[Title/Abstract] OR "cost-benefit"[Title/Abstract]) OR "implementation outcome*"[Title/Abstract])) | 5,228,304 |
| #7 | #5 AND #6 | 716 |
