# Supplementary material for: Implementation strategies and outcomes of school-based programs for adolescent suicide prevention: A scoping review protocol
Source: PLoS One. 2023 May 4;18(5):e0284431. doi: 10.1371/journal.pone.0284431 (PMC10159337; doi:10.1371/journal.pone.0284431)
Supplement: S2 Appendix — (DOCX) [file pone.0284431.s003.docx]

# S2 Appendix: Categories data extraction form

| **Categories** | **Characteristics** |
| --- | --- |
| Study identifiers | First author  Title  Year of publication  Country of study  Article type |
| Intervention description | Type of prevention: universal, indicated, selective, multilevel  Preventive components  Clinical outcomes expected  Interventionist/facilitator  Frequency/Duration  Location/context/practice setting  Target audience  Evaluations  Evidence background |
| Methods: description | Aims and objectives  Design  Context  Targeted ‘sites’  Implementation strategies  Sub-groups |
| Methods: Evaluation | Outcomes  Measures/techniques/tools  Process evaluation  Economic evaluation  Sample characteristics  Methods of analysis |
| Results | Characteristics  Outcomes  Process outcomes  Economic evaluation  Sub-group analysis  Fidelity/adaptation  Contextual changes  Harms  Future research |
